# Murine Gut Microbiota Is Defined by Host Genetics and Modulates Variation of Metabolic Traits

**DOI:** 10.1371/journal.pone.0039191

**Published:** 2012-06-18

**Authors:** Autumn M. McKnite, Maria Elisa Perez-Munoz, Lu Lu, Evan G. Williams, Simon Brewer, Pénélope A. Andreux, John W. M. Bastiaansen, Xusheng Wang, Stephen D. Kachman, Johan Auwerx, Robert W. Williams, Andrew K. Benson, Daniel A. Peterson, Daniel C. Ciobanu

**Affiliations:** 1 Animal Science Department, University of Nebraska, Lincoln, Nebraska, United States of America; 2 Department of Food Science and Technology, University of Nebraska, Lincoln, Nebraska, United States of America; 3 Department of Anatomy and Neurobiology, University of Tennessee Health Science Center, Memphis, Tennessee, United States of America; 4 Laboratory for Integrative and Systems Physiology, Ecole Polytechnique Fédérale de Lausanne, Lausanne, Switzerland; 5 Department of Geography, University of Utah, Salt Lake City, Utah, United States of America; 6 Animal Breeding and Genomics Centre, Wageningen University, Wageningen, The Netherlands; 7 Department of Statistics, University of Nebraska, Lincoln, Nebraska, United States of America; 8 Department of Pathology, John Hopkins University School of Medicine, Baltimore, Maryland, United States of America; University of Illinois, United States of America

## Abstract

The gastrointestinal tract harbors a complex and diverse microbiota that has an important role in host metabolism. Microbial diversity is influenced by a combination of environmental and host genetic factors and is associated with several polygenic diseases. In this study we combined next-generation sequencing, genetic mapping, and a set of physiological traits of the BXD mouse population to explore genetic factors that explain differences in gut microbiota and its impact on metabolic traits. Molecular profiling of the gut microbiota revealed important quantitative differences in microbial composition among BXD strains. These differences in gut microbial composition are influenced by host-genetics, which is complex and involves many loci. Linkage analysis defined Quantitative Trait Loci (QTLs) restricted to a particular taxon, branch or that influenced the variation of taxa across phyla. Gene expression within the gastrointestinal tract and sequence analysis of the parental genomes in the QTL regions uncovered candidate genes with potential to alter gut immunological profiles and impact the balance between gut microbial communities. A QTL region on Chr 4 that overlaps several interferon genes modulates the population of *Bacteroides*, and potentially Bacteroidetes and Firmicutes–the predominant BXD gut phyla. *Irak4*, a signaling molecule in the Toll-like receptor pathways is a candidate for the QTL on Chr15 that modulates Rikenellaceae, whereas *Tgfb3*, a cytokine modulating the barrier function of the intestine and tolerance to commensal bacteria, overlaps a QTL on Chr 12 that influence Prevotellaceae. Relationships between gut microflora, morphological and metabolic traits were uncovered, some potentially a result of common genetic sources of variation.

## Introduction

The gastrointestinal tract contains a vast community of organisms that collectively comprise the microbiota, which is critical for the development of the intestinal epithelium and mucosal immunity as well as contributing digestive metabolic functionalities [1], [2]. The composition of this complex community is established early in life and influenced soon after birth by maternal environment and stochastic exposure to different microbes. Diet, exposure to antibiotics, pathogens, and parasites can also influence compositional features of the microbiota. In a remarkable set of studies, the transition to a low fat diet in overweight humans shifted the gut flora to a composition that resembled that of healthy non-obese matched controls [3], [4]. Conversely, the dissemination of a complex gut flora from overweight animals into the gut of otherwise matched gnotobiotic animals can induce statistically significant weight gains [5], [6].

There is increasing evidence that genetics of the host influence and interact with gut microbiota in various mammals [6], [7], [8], [9], [10], [11]. Early studies focused on enteric pathogens, such as that of Meijerink et al. (2000) who found the adhesion of F18 fimbriated *E. coli* to intestinal mucosa and subsequent susceptibility to swine edema disease to be controlled by fucosyltransferase 1 gene [10]. Several studies in monogenic models have demonstrated the role of innate immune response in altering the composition of mouse gut microbiota and disease susceptibility [6], [12], [13]. For example, deficiency in T-bet (*Tbx2*) promotes a colitogenic microbial population and ulcerative colitis [12], while deficiency in Toll-like receptor 5 (*TLR5*) alters the abundance of microbiota at species level leading to features characteristic of metabolic syndrome [6]. Recently, Benson et al. (2010) using Quantitative Trait Locus (QTL) mapping methods detected genome-wide linkages with the relative abundance of several taxa in the gut of a large murine advanced intercross population [7].

The purpose of the present study is to uncover natural genetic variants present in the host that explain variation in mouse gut microbiota and also explore its impact on obesity and other metabolic phenotypes that affect health. We achieved this by combining the power of next-generation sequencing of gut microbiota with genome-wide linkage analysis and a deep multi-scalar analysis of microbiota across an extensive set of physiological phenotypes in the BXD mouse reference population. BXD is mouse genetic resource characterized extensively at molecular and phenotypic level. This population resulted from the combination of C57BL/6J and DBA/2J genomes [14] and displays important differences in susceptibility to obesity and other morphologic, immunologic, behavioral and metabolic traits. While gut microbiota of C57BL/6J-based genetic resources were previously profiled in various environments, here we introduce the gut microbial profile of DBA/2J, a strain known for its high proportion of body fat mass and predisposition to obesity [15], [16]. Our analysis of gut microbiota of the BXD strains revealed substantial quantitative differences among strains, which can be explained by complex and polygenic influences of the host.

## Results

### Gut microbial profile of the BXD strains is dominated by Firmicutes and Bacteroidetes and displays substantial variability

Pyrosequencing generated 512,646 sequencing reads of the V1–V2 region of the 16S rRNA gene that passed the filtering criteria. An average of 7,651 sequencing reads was obtained per sample. The reads were assigned to different taxonomic units using three approaches. Using a parallelized version of the CLASSIFIER from Ribosomal Database Project (RDP), 97.12% of the sequences were assigned to five phyla groups. Considerable variability was detected at phyla level, with Firmicutes (79.25%) and Bacteroidetes (15.69%) representing the predominant taxa (Table 1). Bacteroidetes is represented at low levels in strains such BXD77 (4.51–7.60%) where Firmicutes accounted for the majority of microbiota (81.11–97.34%). In contrast, in BXD15 the ratio between Bacteroidetes (34.66–40.36%) and Firmicutes (50.47–56.24%) is well balanced. Evidence of Actinobacteria, Proteobacteria and candidate phylum TM7 was detected at very low levels (<1%). Approximately 62% of the sequences were assigned at genus level. *Lactobacillus* was the predominant group (54.1%) with no other genera contributing more than 3.2% of the microbiota. We detected important differences in the abundance of *Lactobacillus* among BXD strains ranging from an average of approximately 22% in BXD12 to 71% in BXD100. Sex did not have a significant effect on the gut microbiota while age had significant effect on three taxa (Bacillales, Staphylococcaceae, *Staphylococcus*) and cage density had effects limited to one taxon (Proteobacteria). The location of the cage (room) had significant effects on 7 taxa that include most of the members of Erysipelotrichi and Clostridia branches. Most of these effects did not reach significance if Bonferoni correction was applied for multiple testing.

**Table 1:** Mean and measures of variability of phyla, genera and Operational Taxonomic Units (OTU) of the BXD strains, their parental lines C57BL/6J and DBA/2J and F1 hybrids (D2B6F1) used in QTL mapping.

Taxa	Mean % (SE)	SD (%)	Min (%)	Max (%)	Median	Samples missing taxa
*Phylum*						
Actinobacteria	0.92 (0.17)	1.38	0.01	8.57	4.40	0
Bacteroidetes	16.00 (1.43)	11.64	0.45	48.58	13.42	0
Firmicutes	78.84 (1.59)	12.89	46.44	97.31	81.28	0
Proteobacteria	0.37 (0.10)	0.88	0.01	6.96	0.18	0
*Genus*						
*Alistipes*	0.30 (0.04)	0.32	0	1.51	0.22	10
*Bacteroides*	1.78 (0.29)	2.31	0	9.54	0.93	8
*Lachnospiraceae*	0.34 (0.05)	0.44	0	2.26	0.22	5
*Lactobacillus*	54.12 (2.34)	18.97	11.80	91.98	54.69	0
*Staphylococcus*	2.78 (0.71)	5.74	0.01	26.96	0.31	2
*OTU*						
*Barnesiella*	0.74 (0.11)	0.91	0	4.48	0.40	4
*Lactobacillus johnsonii*	11.87 (1.05)	8.57	0	32.93	11.34	3
*Lactobacillus murinus*	5.65 (0.75)	6.09	0	28.80	3.74	5

Sequencing reads were also subjected to chimeras removal, alignment and subsequently were clustered using the Complete Linkage Clustering tool of the RDP to generate Operational Taxonomic Units (OTU) (Table 1). The OTUs that have an average of at least 30 counts across BXD samples belong to the genus *Lactobacillus* (*L. johnsonii*, *L. murinus* and *L. intestinalis*), *Staphylococcus* (*S. xylosus* and *S. lentus*) and *Barnesiella. Lactobacillus* OTUs are the predominant species accounting for 25.6% of the OTUs. The most abundant OTU had the highest similarity with *L. johnsonii* accounting for 14.9% of the classified sequences. OTU composition varied substantially among BXD strains. For example, *L. murinus* abundance is negligible in several BXD strains such as BXD96, while in others such as BXD66, the contribution is considerable (28.2%).

In the last approach the top most abundant OTU clusters that accounted for 90% of the reads in the dataset were combined into 158 OTUs using a 97% identity cutoff to eliminate overlap between clusters. The entire dataset was compared to these 158 OTUs and the single best BLAST hit identified for all sequences. This allowed us to assign over an average of 90% of the sequence reads to one of these OTUs (s.d. 5.7%, minimum 71% and maximum 98%) and subsequently assign all the reads to five phyla and approximately 73% of the reads to genera (Table S1).

### Host genetics impacts microbial composition of mouse gut

**Figure 1:**
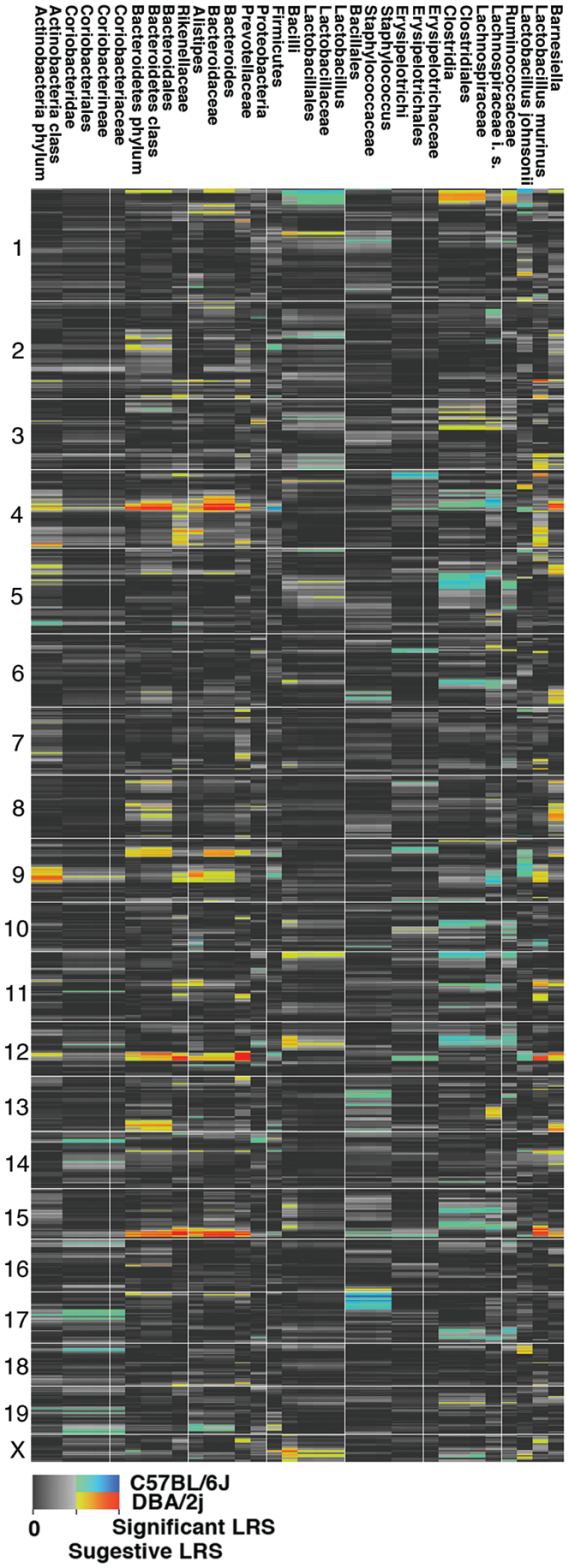
Genome-wide QTL mapping of the gut microbiota in BXD strains. The heat map is represented in “Grey+Blue+Red” in which more intense colors mark chromosomal regions with statistical values associated with high linkage while the spectrum indicates the allelic effect. The blue-green regions are those in which *B* allele is associated with higher trait values, whereas red-yellow regions are those in which the *D* allele is associated with higher trait values. Grey and black regions correspond to insignificant linkage between traits and DNA markers.

QTL analysis of gut microbiota based on CLASSIFIER output revealed five QTL regions (P<0.05) at the genome-wide level for six taxonomic groups (Table 2, Figure 1). Loci associated with significant effects were concentrated on four chromosomes. The QTLs were restricted to a particular taxon, branch or influenced the variation of taxa across phyla. For example, QTLs mapped on Chr 12 have an effect on Prevotellaceae while a QTL mapped on Chr 17 influenced the variation of Bacillales/Staphylococcaceae/*Staphylococcus* branch. In contrast, a QTL located on Chr 4 potentially influenced taxa in different phyla.

**Table 2:** Significant QTLs that influence gut microbial composition in the gut of BXD mice. A positive additive effect indicates that *DBA/2J* alleles increase trait values.

Taxa	Chr	Peak position (Mb)	Confidence intervals (95%)	LRS	Additive effect	Variance explained
*Order*						
Bacillales	17	21.83–23.00	21.71–26.63	19.6	−0.417	0.21
	17	7.75–10.17	7.37–10.44	19.0	−0.412	0.20
*Family*						
Prevotellaceae	12	90.15	78.36–95.80	24.9	0.556	0.27
Rikenellaceae	15	95.15–95.78	92.73–97.39	19.2	0.552	0.23
Staphylococcaceae	17	7.75–10.17	7.39–10.44	19.0	−0.417	0.21
	17	21.83–23.00	21.71–26.66	18.9	−0.416	0.20
*Genus*						
*Bacteroides*	4	87.70–88.04	87.58–95.23	17.9	0.504	0.21
*Staphylococcus*	17	7.75–10.17	5.26–10.43	19.2	−0.414	0.20
	17	21.83–23.00	21.71–26.70	18.9	−0.412	0.20

In contrast, a negative additive effect indicates that *C57BL/6J* alleles increase the trait value.

Gene expression of the gastrointestinal tract and sequence analysis of parental genomes in the QTL regions were used to uncover potential candidate genes that could explain the variation in gut microbiota. Bacteroidetes displayed the largest number of taxonomic units (*Bacteroides*, Prevotellaceae and Rikenellaceae) influenced by host genetics. A QTL located on Chr 15 (LRS = 19.2, 92.73–97.39 Mb) had a significant effect on Rikenellaceae. *Rapgef2* and *Irak4* are two positional candidate genes that harbor non-synonymous SNPs and display important fold difference in expression between parental alleles (1.59 and 1.21×). A similar example was found for the QTL mapped on Chr 12 for Prevotellaceae. One of the candidates for this QTL was *Tgfb3*, an anti-inflammatory cytokine with a potential role in modulating barrier function of the intestine and tolerance to commensal bacteria [17], [18], [19]. *Tgfb3* is differentially expressed in parental strains (1.23–1.45×) with the expression increased by *D2* allele in the jejunum, cecum and ileum.

**Figure 2:**
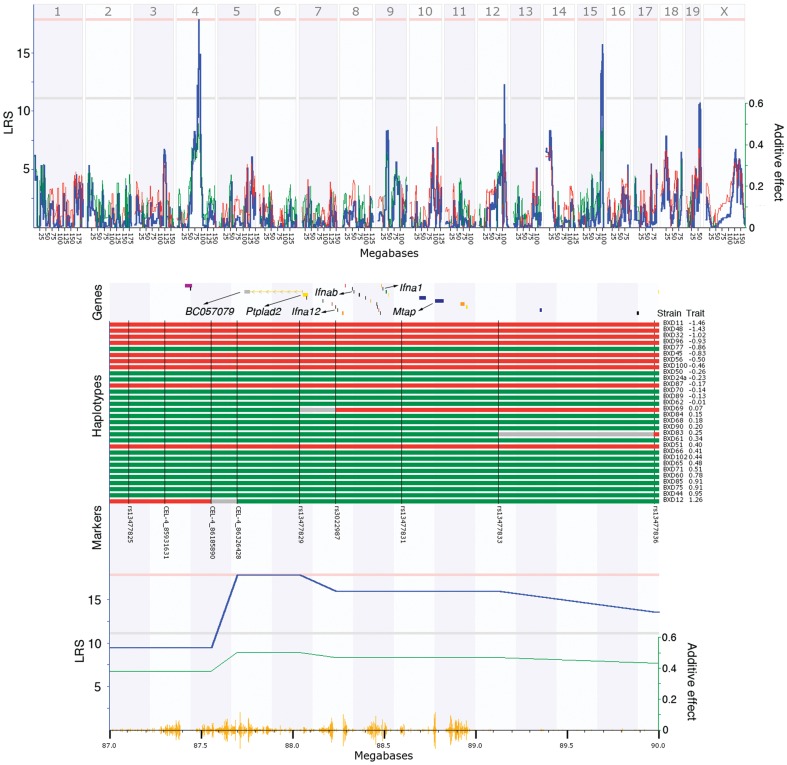
Genome-wide QTL mapping of *Bacteroides* composition in the gut of BXD strains. The Left y axis represents the strength of the linkage between *Bacteroides* composition to different DNA marker intervals on A) each chromosome (blue line) and B) detailed region of the QTL located on Chr 4 (LRS = 20.2, 82.12–95.05 Mb) that explains 27.5% of the composition in *Bacteroides*. The Right y axis represents the additive effect and indicates if the *D* (green line) or *B* (red line) allele contributes to an increase in the abundance of *Bacteroides*. Pink and gray horizontal lines indicates the significant (<0.05) and suggestive (<0.67) QTL threshold. b) *B* haplotype (red) is associated with a reduced level of *Bacteroides* while the *D* haplotype (green) is associated with an increased level of *Bacteroides*. Grey regions represent recombination spots. Yellow seismograph represents the SNP density between the sequence of the parental genomes. The QTL region is rich in genes from interferon family such as *Ifna1*, *Ifna12* and *Ifnab*, all expressed in the cecum of BXD. Additional positional candidates include *Ptplad2*, *BC057079* and *Mtap*.

A QTL that has potential effects across phyla was located on Chr 4. This locus (LRS = 17.9, 87.58–95.23 Mb) explains 21% of the observed variation in the abundance of the genus *Bacteroides* (Figure 2). The same locus was also associated with suggestive effects in Firmicutes (LRS = 13.38, P = 0.27) and Bacteroidetes (LRS = 12.57, P = 0.36) phyla. The C57BL/6J (*B6*) allele (haplotype) from this locus increased the proportion of Firmicutes while the DBA/2J (*D2*) allele increased the proportion of Bacteroidetes. The QTL influenced the variation of the Bacteroidetes/*Bacteroides* branch from phyla to genus, with an increased effect at the tips of the phylogenetic tree (Figure 1). This QTL region is rich in interferon alpha (*Ifna1*, *Ifna2*, *Ifna4* – *Ifna7*, *Ifna9*, *Ifna11*- *Ifna14*, *Ifnab*), beta (*Ifnb1*), zeta (*Ifnz*), and epsilon (*Ifne1*) genes. The expression of this cluster of genes in the gastrointestinal tract was limited to *Ifna1*, *Ifna12*, and *Ifnab*. Considerable fold-difference in expression between parental alleles was detected for *Ifna12* and *Ifnab* with *D2* alleles increasing expression. Additional genes that displayed important fold-differences in expression include *Ptplad2*, *Cdkn2b*, and *Klhl9*. Variation in DNA sequence between parental genomes is relatively limited to the region spanning 87.0 to 89.0 Mb. Analysis of genes located in this interval and expressed in the gastrointestinal tract revealed non-synonymous SNPs in *BC057079* (6 SNPs), *Ptplad2* (1), *Ifnab* (4) and *Mtap* (1). An early stop codon was introduced by a *D2* SNP variant in one of the *Ptplad2* isoforms. Variants of two of the SNPs located in *Ifnab* (rs13477830 and rs32311578) were shared equally in a diversity panel of laboratory mice lines while others (rs28091853 and rs28091852) had very limited variation among lines, with *B6* being the rare allele.

The presence of most of the QTLs was confirmed when sequencing reads were assigned to taxa based on a combination of OTU clustering and CLASSIFIER. The QTL associated with Prevotellaceae was confirmed on the same region of Chr 12 (LRS = 24.09, 88.25–89.93, P = 0.002). The presence of multiple loci mapped adjacently (31.56, 23.32–26.35 and 7.75–10.17 Mb) on Chr 17 was confirmed for Staphylococcaceae and *Staphylococcus* (P<0.02, P<0.06 and P<0.09). The QTL located on Chr 4 (87.7–88.04 Mb) was confirmed having effects close to significance in *Bacteroides* (LRS = 17.7, 87.7–88.04 Mb, P = 0.07) with trends for Bacteroidetes and Firmicutes (P<0.42). Suggestive effects were also confirmed for the QTL located on Chr 15 (92.84–94.02 Mb) for Rikenellaceae (LRS = 14.84, P = 0.20).

A major source of variation of the gut microbiota in BXD is represented by *Lactobacillus*. One of the potential sources of this variation is a suggestive QTL mapped on Chr 10 (LRS = 15.3, 111.13–111.84 Mb, P = 0.054) for an OTU similar to *L. murinus* with *B6* allele increasing its abundance. The effect of this QTL is not restricted to *L. murinus* but has a potential effect across phyla. A QTL located in the same position potentialy influences the variation in abundance of *Alistipes* (LRS = 13.84, 111.13–111.84, P = 0.23), a member of Bacteroidetes. A strong correlation was detected between *L. murinus* and *Allistipes* (r = 0.81, p<0.0001), potentially as a result of common or linked genetic sources of variation. Important fold-differences in cecum expression between parental alleles of the genes located in this QTL area were found for *Bbs10*, *Krr1* and *Nap1l*. If the sources of these differences in expression are caused by the same regulatory elements, we assumed same modulation mechanisms and direction of effects across BXD tissues [20]. We found consistent evidence of *cis*-regulation of gene expression for *Nap1l1* in hippocampus [21], whole brain [22] and liver [23] data sets and for *Bbs10* in striatum [24] and retina [25]. The direction of the additive effect was consistent across tissues and in agreement with the expression of parental alleles in the gastrointestinal tract. Presence of non-synonymous DNA sequence variants was limited to *Bbs10*, a chaperonin-like protein and one of the major genetic sources of Bardet-Biedl syndrome in humans with characteristics including postnatal obesity and diabetes among others [26]. The *B6* alleles of the *Bbs10* are the most common across a diverse set of inbred strains.

### Relationships exist between gut microflora, morphologic, and metabolic traits

The BXD strains displays important phenotypic variation for anatomic, developmental and metabolic traits (Figure S1 and S2). Several BXD strains (BXD28, BXD16, and BXD34) are characterized by large weight gains (0.58 to 1.08 g/week of 13 to 20 weeks-old males) while others (BXD69, BXD56, and BXD98) are characterized by lack of or limited growth (−0.10 to 0.08 g/week) (Figure S1). Feed intake and activity level are two of the sources that can explain some of the differences in body weight among BXD strains (P<0.005). Feed consumption varies among strains ranging from less than 1.30 g (BXD16, BXD103, and BXD 31) to more than 3 g of feed consumed per 24 hr (BXD69, BXD45, BXD31, and BXD68). Activity level during daytime ranges from 1155.8–1304.8 fine movements/hour in extremely active strains (BXD8, BXD75, BXD18, and BXD86) to 424.8–467.1 movements/hour in more sedentary strains (BXD96, BXD71, and BXD69). To examine the sources that could influence susceptibility to metabolic syndrome in BXD, we analyzed the relationship between weight gains and body composition. We found moderate correlations between average weekly gain and fat (r = 0.48, P<0.0001) and lean mass (r = −0.45, P<0.001). Variation in body fat composition ranged from 9.65% (BXD20, s.e. = 0.99) to 26.18% (BXD92, s.e. = 2.90) with an average of 15.67% (s.e. = 0.33) (Figure S2). These differences in body fat composition could explain approximately 13% (P<0.001) of the blood glucose level expressed as area under the curve following a glucose tolerance test.

Gut microbial profiles were subjected to principal component analysis (PCA) to determine if variation in gut microbiota could explain some of the differences in weight, body composition and susceptibility to diabetes. The presence and magnitude of these relationships were influenced by sex. The first eigenvector explained 24.3% of the variation of classified taxa and is associated with weekly body weights of 14 to 20 weeks-old females (P<0.05) but not in males. Phenotypic correlations were also estimated between the abundance of each taxonomic group and a set of 143 metabolic traits available for BXD strains [15]. The number of BXD strains that have both microbiota and metabolic traits profiled varied by trait from 9 to 21 strains. Consistent relationships in both sexes were estimated between *L. johnsonii* and several morphologic and metabolic traits. Increased abundance of *L. johnsonii* in females was associated with increased white adipose (r = 0.68, P<0.01) and body fat mass (r = 0.57, P<0.05) and suggestive relationships with body weight (r = 0.39, P<0.16) and lean mass (r = −0.42, P<0.15). Similarly, increased abundance of *L. johnsonii* in males was associated with increased body weight (r = 0.52, P<0.05), lean (r = −0.49, P<0.05) and fat mass (r = 0.45, P = 0.05) and suggestive relationships with daytime activity (r = −0.46, P = 0.07) and white adipose mass (r = 0.30, P<0.20). Total body fat (r = −0.71, p<0.005), white fat (r = −0.57, p<0.05) and body lean mass (r = 0.65, p<0.05) of BXD females were associated with the ratio of Firmicutes to Bacteroidetes (Figure S3). Increased level of Bacteroidetes (r = 0.68, p<0.01) and reduced level of Firmicutes (r = −0.75, p<0.005) indicated high body fat composition. The direction of these relationships was consistent but not significant in males. False discovery rate of the significant relationship presented (p<0.05) vary from 0.09 to 0.47 but the distribution of P values clearly deviates from the null hypothesis.

## Discussion

The random segregation and recombination of the C57BL/6J and DBA/2J genomes into BXD strains generated substantial variability for key physiological phenotypes including feed intake, activity level, energy expenditure, body composition, and susceptibility to metabolic syndrome and other diseases. In this study we have extended the scope of phenotyping by studying host genetics and other sources that underlie variation of gut microbiota in BXD. This is the first study that has carried out a deep multiscalar analysis of the complex gut microbiota in combination with a set of physiological phenotypes.

Murine gut microbiota is influenced by several factors including diet, host-genetics, age, environment, and caging history [7]. Molecular profiling of the gut microbiota revealed important quantitative differences in microbial composition among BXD strains. As in the previous studies, Firmicutes and Bacteroidetes represented the predominant groups of gut microbiota [7], [27], [28] with the rest of the phyla (Actinobacteria, Proteobacteria and TM7) having a very limited contribution but substantial variation among strains.

Linkage analysis of the data generated by deep sequencing of microbial DNA evidenced the influence of host genetics on the composition of gut microbiota in two months-old naive BXD strains. In our previous work using *cis* expression QTL as empirical measures of mapping precision we showed that BXD data sets of a similar size can provide sufficient precision (∼1 kb from the source gene to QTL peak) to map QTLs with LODs scores of 3–4 [19]. The QTLs identified in this study were restricted to a particular taxon, branch, or influenced the variation of taxa across phyla. Gene expression of the gastrointestinal tract and sequence analysis of parental genomes in the QTL regions uncovered several candidate genes that have the potential to alter gut immunological profiles and subsequently impact gut microbial composition. A QTL mapped on Chr 4, located in a region rich in interferon genes, influenced the variation of *Bacteroides* and potentially Bacteroidetes and Firmicutes, the predominant BXD phyla. *Tgfb3*, a cytokine with a potential role in modulating the barrier function of the intestine and tolerance to commensal bacteria [17], [18], [19], represents one of the candidate genes for a QTL mapped on Chr 12 that influenced Prevotellaceae. *Irak4*, a signaling molecule in Toll-like receptor pathways [29], represents a potential source for the QTL mapped on Chr 15 that influenced Rikenellaceae. IRAK4 interacts with MYD88 adapter protein, which is used by several TLR in host defense [30] and control of commensal bacteria [31]. Mutations in *IRAK 4* and *MYD88* in humans impair some of the TLR pathways and are associated with predisposition to pyogenic bacterial infections [29]. Loss of *Myd88* in mice impacts the innate immune response to pathogens and influences gut microbiota by increasing the composition of three bacterial families including Rikenellaceae [13]. This microbial profile can reduce the progression of autoimmune diabetes in a *Myd88*-independent manner [13]. We found a strong correlation between the expression of *Irak4* and *Myd88* across 25 different tissues in the parental strains of BXD (r = 0.79, P<0.0001) that indicates a tight functional relationship between these genes. The same or closely linked alleles inherited from DBA/2J are associated with a lower expression of *Irak 4* as well as a higher proportion of Rikenellaceae. Recently, Benson et al. found *Irak3*, a modulator of the MYD88-dependent TLR2 pathway, as a potential candidate for a QTL on Chr 10 that influence Coriobacteriaceae and *Lactococcus* [7].

*Lactobacillus* was the predominant taxa at genus level and a substantial contributor to the quantitative differences in gut microbiota among BXD strains. The source of variation could have important implication since *Lactobacillus* is known to have immunomodulating properties [32]. The *B6* allele of the suggestive QTL mapped on Chr 10 increased the abundance of *L. murinus*. A QTL located in the same region was found by Benson et al. to influence *Lactococcus* (order Lactobacillales) [7]. This genus is closely related to *Lactobacillus*, which was associated with low counts in the population used in Benson et al. study.

It is expected that a specific gut immunological profile has the potential to alter gut microbiota [5], [6] favoring a microbiome that possesses increased efficiency of extracting energy from food [4], [8], [33]. Association analyses between gut microbial composition, especially the ratio between Firmicutes to Bacteroidetes, and obesity generated contradictory results [27], [33], [34], [35], [36], [37]. One of the important roles of intestinal microorganisms is to break down the dietary fiber and starch incompletely hydrolyzed by intestinal enzymes of the host [38], [39], [40]. Short chain fatty acids (SCFA), specifically acetate, propionate and butyrate, are the main fermentation products of fiber digestion and represent an additional source of energy since they can be used for lipid or glucose *de novo* synthesis [41], [42]. Changes in the SCFA profiles were associated with changes in gut microbiota [37], [43] as well as with variation in body mass index [37]. Propionate [36], [43] and butyrate [43] are favored in overweight and obese subjects, suggesting an important role of SCFA metabolism in obesity. Propionate is absorbed and delivered to hepatocytes where is used as a precursor for gluconeogenesis, lipogenesis and protein synthesis [42], [44], [45]. The *D2* alleles from two QTLs identified in this study increased the abundance of *Bacteroides* and Prevotellaceae, known propionate producers [37], [46]. DBA/2J has a substantial higher proportion of body fat mass than C57BL/6J and is predisposed to obesity [15].

The position of the QTL located on Chr 4 that increased the proportion of *Bacteroides*, coincides with a QTL (*Adip11*, peak at 88.710 Mb) associated with inguinal and retroperitoneal fat pads mass in a F2 cross between SM/J x NZB/BlNJ (n = 513) mouse strains following 16-weeks of feeding with atherogenic diet [47]. SM/J is characterized by higher adiposity compared to NZB/BlNJ. Individual fat pad weights are not available in BXD and there are no QTL located on Chr 4 associated with whole fat body mass. However, most of the fat in the SM/J x NZB/BlNJ F2 cross was represented by mesenteric and gonadal pads (68 to 86% in parental strains of SM/J x NZB/BlNJ) which were not influenced by the same QTL located on Chr 4 [47]. Using the genotypes generated by Mouse Diversity Array [48] we compared the parental haplotypes in the peak area of the Chr4 QTL for *Bacteroides* (Figure 2). As expected, we found *SM/J* haplotype very similar to *DBA/2J* while *C57BL/6J* is more similar to *NZB/BINJ*, suggesting that a QTL influencing gut microbial profile could impact energy metabolism and affect fat pads weight. This QTL region is rich in genes from the interferon family. Differences in cecum gene expression between parental alleles of the BXD were found for two members of type I interferon (IFN-α) family of cytokines, *Ifna12* and *Ifnab*, with the later being rich in non-synonymous SNPs. There is limited information about the biological role of *Ifnab* but detailed analysis of *Ifna12* indicated that it has functional characteristics of type I IFN-α proteins [49]. In combination with other mediators such as TNF-α and IL-12, IFN-α is produced in response to microbial products and modulate innate and adaptive immune response [50], [51]. In mice, type I IFN consists in multiple IFN-α subtypes and one IFN-β and IFN-ω, all binding a ubiquitously expressed heterodimeric receptor – IFNAR [52]. IFN-α/β genes are involved in Toll-like receptor, RIG-I –like receptor, Jak-STAT signaling pathways and in natural killer cell mediated cytotoxicity and as a result could impact the balance between various microbial communities in the gut. The role of IFN-α/β in immune function and intestinal homeostasis was underlined by Katakura et al. [53]. Using a murine model they discovered a protective role of IFN-α/β in experimental colitis as a result of cross-regulation mechanisms with TNF-α.

While variation in body weight and susceptibility to obesity are known to be strongly influenced by genetic variation (*h^2^* = 0.40–0.80) [54], [55], [56], recent genome-wide association studies uncovered a relatively modest fraction of genetic variants that could explain variation of body mass index [57], [58], predisposition to obesity [57], [59], [60], [61], [62] and fat distribution [63], [64]. Variation in gut microbiota and complex relationships with host genetics can represent unaccounted sources of differences for physiological phenotypes including susceptibility to obesity.

The BXD reference population has been used since 1973 [65] to characterize genetic factors that control variation of both Mendelian and complex traits [66]. Recently, BXD strains have undergone extensive phenotyping and molecular characterization including deep sequencing of the parental lines and identification of approximately 4.9 million SNPs and hundreds of thousands of insertion/deletions and copy number variants [67]. This deep compendium of sequence polymorphisms represents a genetic resource that models some aspects of sequence variation in human populations. In this study, the BXD population was used to detect and quantify genetic factors that may have a significant influence on the variation of gut microbiota. We have demonstrated that host-genetics is complex and involves many loci. These differences in microbial composition could impact susceptibility to obesity and other metabolic traits. Functional analysis of gut microbiota and characterization of the relationships with host-genotype has important implications to human health and agriculture. The gut microbial composition can be temporarily altered through dietary interventions tailored to host genotype, ultimately mitigating the effects of unfavorable alleles and inducing profiles that promote human health. Genetic variants that influence gut microbiota may also be used in selection programs of livestock to improve feed efficiency, disease resistance, and to reduce dissemination of pathogens associated with zoonotic diseases such as *E.coli* O157:H7 or *Salmonella*.

## Materials and Methods

### Animals, animal care and sample collection

Fecal samples were collected in less than 24 hours period from all 30 BXD strains used in mapping. Fecal samples were also collected from the parental lines of BXD strains, C57BL/6J and DBA/2J and from F1 hybrids (D2B6F1). Fecal pellets were collected separately from two months-old naive males and female by pooling samples from each cage and storing at −80°C. The mice were housed at the University of Tennessee Health Science Center (UTHSC) in a specific pathogen-free environment at 20–24°C with a light/dark cycle of 14/10 hr. and *ad libitum* access to food and water. Male and female littermates were housed in separate cages. The cages were located in four different rooms of the same facility with a relatively balanced ratio of cages allocated to each sex per room and with an average of three mice per cage (Table S2). The fat source of the diet was derived from soybean oil providing approximately 17% of energy and containing 0.8% saturated, 1.3% monosaturated, and 2.9% polyunsaturated fatty acids (diet 7019, Harlan Teklad). All animals were kept in accordance with guidelines set by the NIH Guide for the Care and Use of Laboratory Animals and under the prevue of the Institutional Animal Care and Use Committee (IACUC) at the UTHSC. The IACUC at the UTHSC specifically approved the study (Permit No. 680).

### Pyrosequencing of the Gut Microbial DNA

DNA was extracted from a pool of approximately 0.10 mg of fecal pellets collected from each cage using QIAmp DNA stool Mini Kit (Qiagen) following cell lysis as described previously [68]. The 16S rRNA gene was amplified using primers targeting the V1–V2 region and containing bar-coded adapters. The forward primer used was 5′-AGAGTTTGATCMTGGCTCAG-3′and the reverse primer was 5′-CTGCTGCCTYCCGTA-3′. Sequencing of the products was performed from one end of the amplicons using Roche 454 GS FLX Titanium chemistry.

### Pyrosequencing data analysis

Quality control of the raw sequences was based on a filtering protocol that excludes sequences that are short, missing barcodes, have high nucleotide ambiguities (>2), or low average quality scores (<25). The reads were distributed to each sample based on barcodes. The reads were assigned to different taxonomic units using three approaches:

- A parallelized version of CLASSIFIER from the Ribosomal Database Project (RDP) was used to assign sequences to taxonomic groups as previously described [7]. The reads in each taxonomic bin were normalized to the proportion of the total number of reads per each sample.
- After quality control procedures, sequences were submitted to the open source software package Quantitative Insights into Microbial Ecology (QIIME) [69] to remove chimeric sequences using the Chimera Slayer method. Sequences were subjected to alignment by ALIGNER and subsequently clustered using the Complete Linkage Clustering tool of the RDP to generate Operational Taxonomic Units (OTU). OTUs are defined units that include sequences with similarities that exceed 97%. OTUs were binned by sample using QIIME [69], and those whose average was above 30 sequences per OTU were selected for QTL mapping.
- In the third approach representative sequences were selected from the originally identified OTUs that accounted for 90% of assigned reads in the dataset. These reads were re-clustered into 158 non-overlapping OTUs using a 97% identity cutoff. The entire dataset was then compared to the database of these 158 non-overlapping OTUs and the single best BLAST hit identified for all sequences in the dataset using BioEdit Blastall function. Alignments of 200 bp and greater than 97% identity were tabulated for each OTU. Each OTU representative sequence was assigned a taxonomy using RDP Seq Match and the counts of those of the same Genus, Family or Phylum were added together for QTL mapping.

### QTL mapping

Proportion values of the microbial profile of the taxa that had at least an average of 20 counts/sample and not more than 10 samples lacking reads were log10 – transformed. Samples lacking sequencing reads for a given taxon were assigned a value of 0.5 divided by the total number of reads of the sample, which was log10 – transformed [7]. The influence of sex, age and cage density on gut microbial composition was tested using a linear-model. A linear model that included cage location was employed to generate residuals for all taxa. Cage density and age was incorporated in the model when significant. We computed the regression between the genotypes of 3,785 informative markers and average of the proportion values of males and females samples for each strain using QTL Reaper to map QTLs for gut microbiota as previously described [21]. Genome-wise empirical P values were obtained by permuting data for each phenotype (proportion values) randomly between 1,000 and 1,000,000 times. Confidence intervals for QTLs were obtained by likelihood support interval (1.5-logarithm of the odds, LOD) as described [70].

### Phenotypes

Correlations between relative abundance of gut microbiota and a BXD set of physiological phenotypes stored in GeneNetwork database (www.genenetwork.org) were computed using Pearson product-moment correlations. This set includes143 traits associated with energy metabolism, blood chemistry, morphology and cardiology, that were generated as part of a large and systematic phenotyping study of 17 to 43 BXD strains by Koutnikova et al. (2009, GeneNetwork Record ID: 11017, 11287, 12076, 12821–12960) [16]. False discovery rates of the multiple comparisons were estimated for each taxon based on the p-values resulted from correlation estimates using the R package qvalue [71].

### Gene expression

Gene expression profile was employed to uncover genes that are expressed in the gastrointestinal tract and are potential candidates for the QTL. Measurement of mRNA expression was performed by Illumina Mouse WG-6 v2.0 array using tissues collected from the gastrointestinal tract of C57BL/6J and DBA/2J males including esophagus, stomach, duodenum, jejunum, ileum, cecum, and ascending colon as previously described [19]. Illumina microarray data was normalized using the vendor's rank invariant method (www.illumina.com). Gene expression and expression QTL data [20]–[24] can be accessed from www.genenetwork.org.

## Supporting Information

Figure S1 **Average body weight of BXD strains between 13 to 20 weeks of age.**
(TIF)

Figure S2 **BXD display important difference for morphological and metabolic traits.** A) Proportion of body fat composition, B) Area under the curve for glycemia following a glucose tolerance test, C) Total cholesterol and, D) Triglyceride levels.
(TIF)

Figure S3 **A negative relationship was detected between the ratio of Firmicutes to Bacteroidetes and body fat mass (g) of 19 weeks-old BXD females (r = −0.71, p<0.005).**
(TIF)

Table S1 **Mean and measures of variability of the gut microbiota of the BXD strains, their parental lines (C57BL/6J and DBA/2J) and F1 hybrids (D2B6F1) based on the combination of OTU clustering and CLASSIFIER.**
(XLSX)

Table S2 **Cage density and the distribution of BXD mice in experimental rooms.**
(XLSX)
