# Supplementary figures and images for: Murine Gut Microbiota Is Defined by Host Genetics and Modulates Variation of Metabolic Traits
Source: PLoS One. 2012 Jun 18;7(6):e39191. doi: 10.1371/journal.pone.0039191 (PMC3377628; doi:10.1371/journal.pone.0039191)

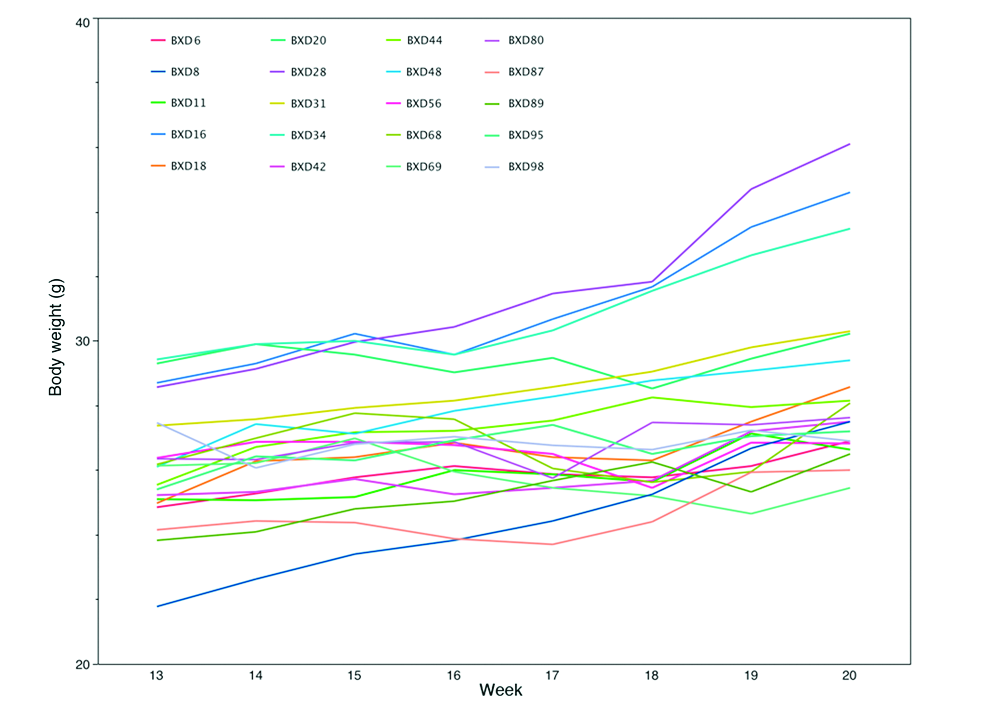

Supplement: Figure S1 — Average body weight of BXD strains between 13 to 20 weeks of age. (TIF) [file pone.0039191.s001.tif]

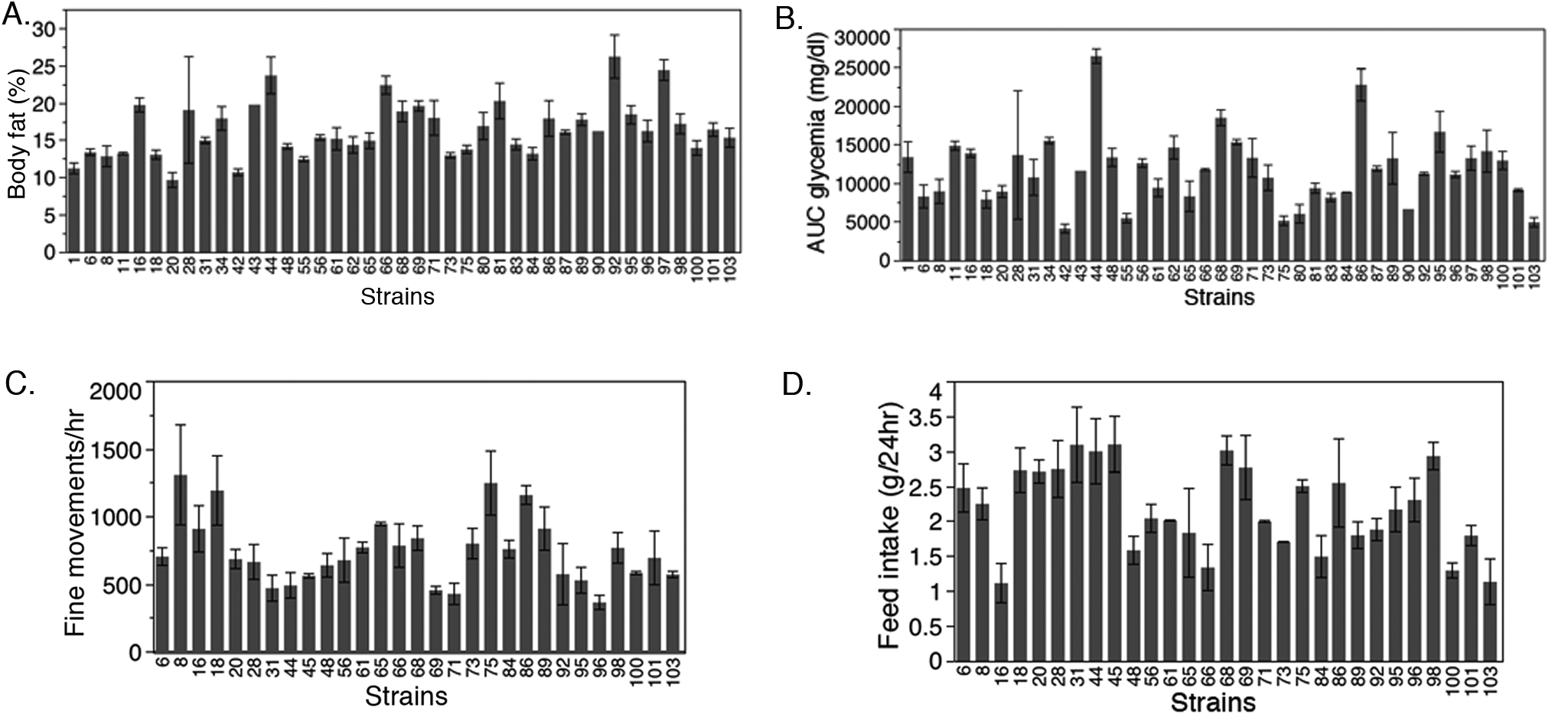

Supplement: Figure S2 — BXD display important difference for morphological and metabolic traits. A) Proportion of body fat composition, B) Area under the curve for glycemia following a glucose tolerance test, C) Total cholesterol and, D) Triglyceride levels. (TIF) [file pone.0039191.s002.tif]

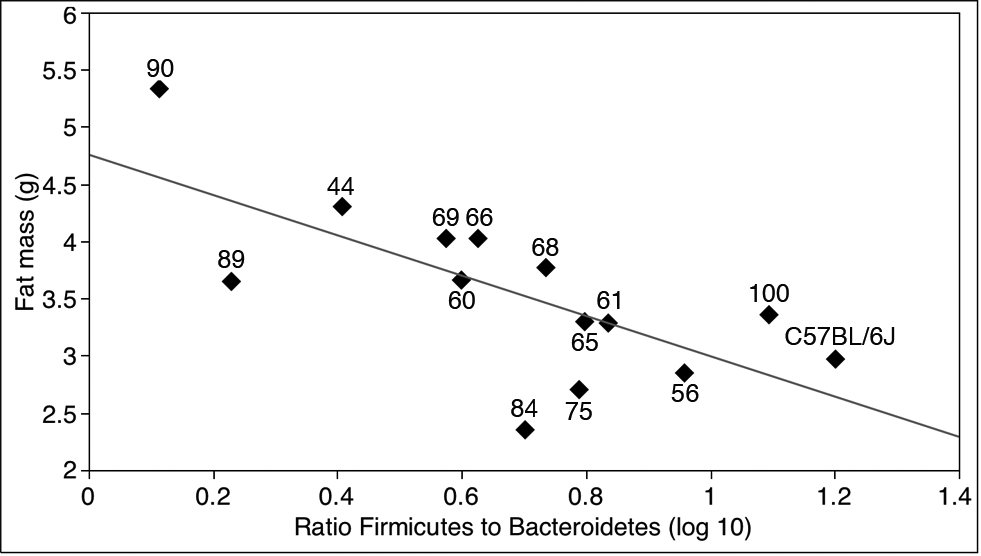

Supplement: Figure S3 — A negative relationship was detected between the ratio of Firmicutes to Bacteroidetes and body fat mass (g) of 19 weeks-old BXD females (r = −0.71, p<0.005). (TIF) [file pone.0039191.s003.tif]
